# Metabolic Syndrome as a Risk Factor of Endometrial Cancer: A Nationwide Population-Based Cohort Study of 2.8 Million Women in South Korea

**DOI:** 10.3389/fonc.2022.872995

**Published:** 2022-06-16

**Authors:** HyunA Jo, Se Ik Kim, Wenyu Wang, Aeran Seol, Youngjin Han, Junhwan Kim, In Sil Park, Juwon Lee, Juhwan Yoo, Kyung-Do Han, Yong Sang Song

**Affiliations:** ^1^ Cancer Research Institute, College of Medicine, Seoul National University, Seoul, South Korea; ^2^ World Class University (WCU) Biomodulation, Department of Agricultural Biotechnology, Seoul National University, Seoul, South Korea; ^3^ Department of Obstetrics and Gynecology, Seoul National University College of Medicine, Seoul, South Korea; ^4^ Interdisciplinary Program in Cancer Biology, Seoul National University, Seoul, South Korea; ^5^ Department of Agricultural Biotechnology, Seoul National University, Seoul, South Korea; ^6^ Department of Biomedicine & Health Science, The Catholic University of Korea, Seoul, South Korea; ^7^ Department of Statistics and Actuarial Science, Soongsil University, Seoul, South Korea

**Keywords:** menopause, endometrial cancer, metabolic syndrome, incidence, cohort

## Abstract

**Background:**

A positive relationship was reported between metabolic syndrome and the risk of endometrial cancer. Studies on the relationship between metabolic syndrome and endometrial cancer have been mainly conducted in post-menopausal women. We aimed to investigate the risk of endometrial cancer according to metabolic syndrome and menopausal status using the Korean nationwide population-based cohort.

**Methods:**

We enrolled 2,824,107 adults (endometrial cancer group; N = 5,604 and control group; N= 2,818,503) from the Korean National Health Insurance Service checkup database from January 1 to December 31, 2009. The median follow-up duration was 8.37 years. Metabolic syndrome was diagnosed as having at least three of the following five components: abdominal obesity, hypertriglyceridemia, low levels of high-density lipoprotein cholesterol, raised blood pressure, and hyperglycemia. Multivariate Cox proportional hazard models were used to calculate hazard ratios (HRs) with 95% confidence intervals (CIs) to estimate endometrial cancer risk.

**Results:**

The endometrial cancer risk was higher in the metabolic syndrome group than that in the non-metabolic syndrome group (HR, 1.362; 95% CI, 1.281–1.449). The association between metabolic syndrome and endometrial cancer risk was significant in the premenopausal subgroup (HR, 1.543; 95% CI, 1.39–1.713) and postmenopausal subgroup (HR, 1.306; 95% CI, 1.213–1.407). The incidence of endometrial cancer was more closely related to metabolic syndrome components in the pre-menopausal subgroup than those in the post-menopausal subgroup (for waist circumference, blood pressure, triglycerides and high-density lipoprotein cholesterol, all *p* for interaction <0.0001 respectively, and for fasting blood glucose, *p* for interaction 0.0188). The incidence of endometrial cancer positively correlated with the number of metabolic syndrome components (log-rank *p <*0.0001).

**Conclusion:**

Our large population-based cohort study in Korean women suggests that metabolic syndrome and its accumulated components may be risk factors for endometrial cancer, particularly in the pre-menopausal women.

## Introduction

Endometrial cancer is the sixth most common female cancer, and the incidence rate has been rising rapidly in the past several decades worldwide (1). The incidence of endometrial cancer is higher in developed countries than in developing countries (2). From 2012 to 2020, the incidence of endometrial cancer has doubled in the United States; it increased from 32,000 cases to 65,620 cases (3, 4). In Korea, the incidence of endometrial cancer gradually increased between 1999 and 2020 (5, 6). The western lifestyle, increase in the obese population, and low fertility rate are all presumed to be possible contributors to the increasing incidence of endometrial cancer (7, 8).

Metabolic syndrome, a pathologic condition characterized by abdominal obesity, insulin resistance, dyslipidemia, hypertension, and hyperglycemia, has emerged as one of the most pressing public health issues worldwide (9, 10). With the growing population of obese adults, the frequency of metabolic syndrome has increased dramatically in recent decades (11). In Korea, the prevalence of metabolic syndrome increased from 21.6% in 2007 to 22.9% in 2018 (12).

Obesity is one of the most essential features of metabolic syndrome and is closely related to cancer incidence and high mortality (13, 14). Several studies had demonstrated that metabolic syndrome was associated with the development of various malignancies, including liver, colorectal, ovarian, breast, and thyroid cancers (15–19). A positive relationship was also reported between metabolic syndrome and the risk of endometrial cancer (20–23). However, despite the recent trend of increasing obesity and metabolic syndrome rates among young women worldwide (24–27), most studies related to endometrial cancer and metabolic syndrome had been conducted mainly on post-menopausal women. Only limited evidence is available on the risk of endometrial cancer according to the combination of metabolic syndrome and menopause status.

Therefore, we aimed to investigate the association between metabolic syndrome and the risk of endometrial cancer according to the menopausal status using the Korean nationwide population-based cohort.

## Methods

### Data Source and Study Population

This nationwide population-based cohort study was carried out after an approval from the Institutional Review Board of Seoul National University Hospital (No. 1811–048–983). We used a customized database from the National Health Insurance Service (NHIS) of Korea. As all data were anonymized in accordance with the NHIS’s confidential guidelines, there was no need for prior consent.

The NHIS is the Korea’s single public healthcare system, offering universal and affordable medical care to most of the Korean population. Also, all insured adults are given a standardized health screening related to metabolic syndrome, including physical measurement, blood pressure measurement and blood test every two years by the NHIS. In Korea, the national cancer registration project is underway, and when cancer is pathologically diagnosed, it is entered into the NHIS database. For the study purpose, we created a customized database by merging the NHIS Medical Check-up DB, which contains 2009 NHIS health exams and cancer screening questionnaire results, and the NHIS claim DB. Patients diagnosed with endometrial cancer were identified using the International Classification of Disease, 10th Revision (ICD-10) code, C54-55.

**Figure 1:**
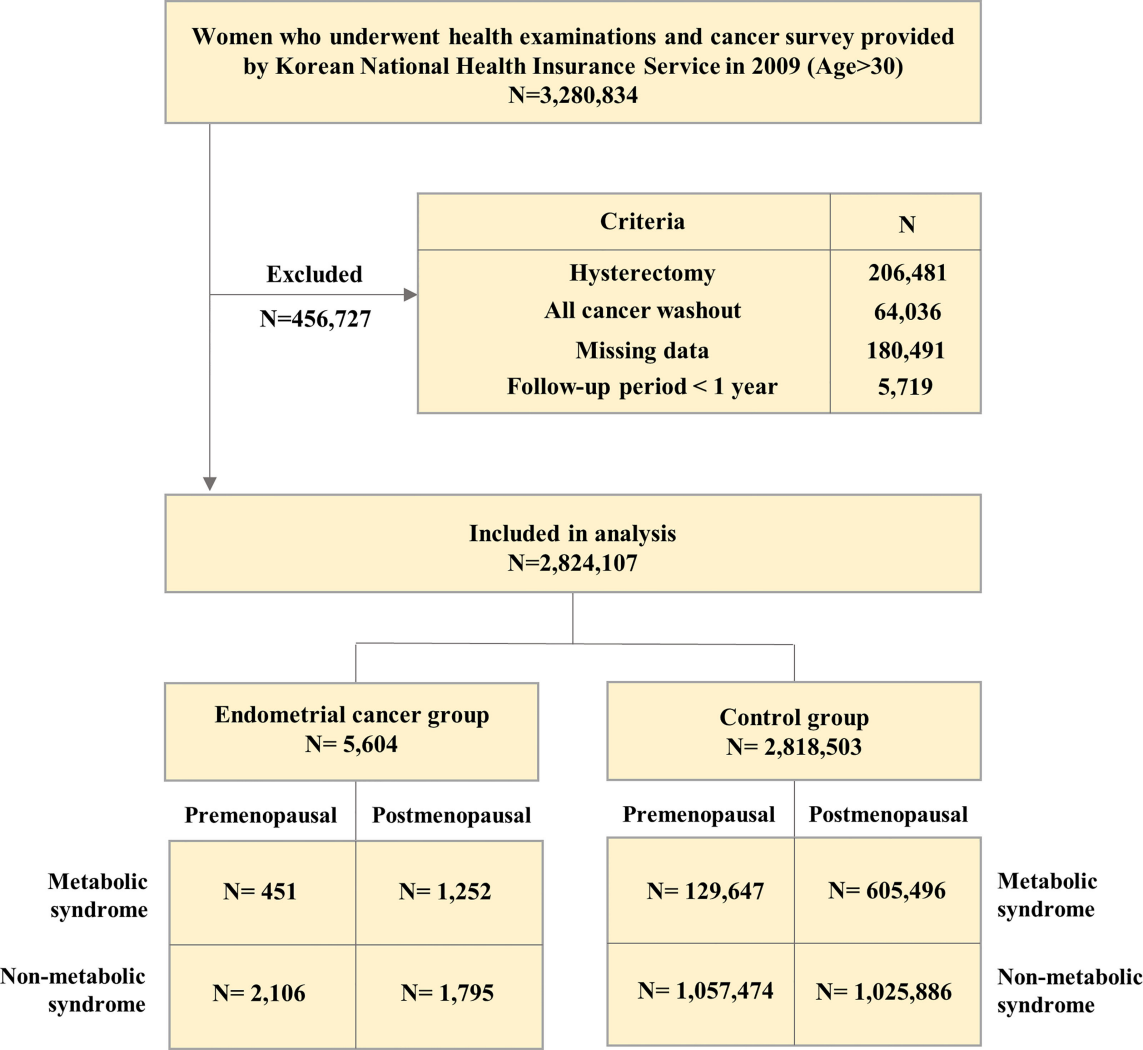
Flow diagram of the search strategy and study selection.

From the customized database, we identified women aged ≥ 19 years and who have been examined and completed the cancer screening questionnaire between January 2009 and December 2009 (*N* = 3,280,834). Among them, we excluded the following women: those who had received hysterectomy (*N* = 206,481); those who had been diagnosed with cancer other than endometrial cancer (*N* = 64,036); and those with missing data (*N* = 180,491). We also excluded those whose follow-up period was less than one year to ensure a causal relationship and reduce detection bias (*N* = 5,719). Consequently, the study population included 2,824,107 women and were observed until the development of endometrial cancer or until December 31, 2018, whichever came first. To investigate the association between metabolic syndrome and endometrial cancer incidence, each population was divided into two groups according to the presence or absence of metabolic syndrome (**Figure 1**). To confirm the association between metabolic syndrome and endometrial cancer incidence in relation to women’s menopausal status, each group (endometrial cancer and control group) was further divided into pre-and post-menopausal subgroups.

### Data Collection

Demographic characteristics, including smoking, alcohol consumption, physical activity, and menopausal status, were obtained from the self-reporting survey. Income levels were divided at the lower 20% and smoking status was divided into three categories: those who have never smoked more than 5 packs of cigarettes in their lives (non-smokers), those who have smoked in the past but do not do it now (ex-smokers), and those who continue to smoke to this day (current smokers). Data on women’s duration of smoking and the cessation data were not available because the limited available variables based on the limited data in the current database. The alcohol consumption category was classified into three groups: non, mild (<30 g of alcohol a day), and heavy alcohol consumption (≥30 g of alcohol a day) according to the amount of alcohol consumed (28). The level of strenuous exercise done for at least 20 minutes a week (none, 1–4 times/week, or 5 times/week) was used to categorize physical activity. Women who were still menstruation were categorized as pre-menopausal ones, while women who had ceased menstruating for a year were classified as post-menopausal ones. Besides, comorbidities such as hypertension (ICD-1-CM codes I10e13 and I15 and antihypertensive drugs), diabetes mellitus (ICD-10-CM codes E11eE14 and oral antidiabetic agents or insulin), and dyslipidemia (ICD-10-CM code E78 and dyslipidemia agents) were investigated using ICD-codes, and prescription medication records.

The determination of metabolic syndrome was dependent on a health examination provided by NHIS, which includes anthropometric and laboratory measurements. The participants’ height, weight, and waist circumference (WC) were all measured, and the body mass index (BMI) was calculated by dividing weight (kg) by height (m) squared. For systolic and diastolic blood pressure (SBP and DBP, respectively) participants were rested at least five minutes before measurement and measured in a seated position. Blood sampling was conducted after overnight fasting, and the following variables were measured: glucose, total cholesterol, triglycerides (TG), high-density lipoprotein cholesterol (HDL-C), and low-density lipoprotein cholesterol (LDL-C) (**Table 1**).

**Table 1:** Baseline characteristics of the study population.

Number of participants	Endometrial Cancer		*p*-value
	No (N=2,818,503, %)	Yes (N=5,604, %)	
Age, years	54.03 ± 11.48	53.28 ± 9.95	<.0001
BMI, kg/m^2^	23.72 ± 3.19	24.54 ± 3.58	<.0001
WC, cm	77.89 ± 8.83	79.46 ± 9.11	<.0001
Low income**^a^**	695,425 (24.67)	1,382 (24.66)	0.9826
Smoking			0.0004
Never	2,688,742 (95.4)	5,397 (96.31)	
Ex-smoker	41,582 (1.48)	83 (1.48)	
Current smoker	88,179 (3.13)	124 (2.21)	
Alcohol consumption**^b^**			0.2113
None	2,259,378 (80.16)	4,544 (81.08)	
Mild	536,316 (19.03)	1,019 (18.18)	
Heavy	22,809 (0.81)	41 (0.73)	
Regular Exercise**^c^**	488,213 (17.32)	1,037 (18.5)	0.0194
Hypertension	912,278 (32.37)	1,933 (34.49)	0.0007
Diabetes mellitus	255,304 (9.06)	547 (9.76)	0.0671
Dyslipidemia	683,860 (24.26)	1,431 (25.54)	0.0265
SBP, mmHg	121.85 ± 16.06	122.67 ± 16.18	0.0001
DBP, mmHg	75.19 ± 10.28	75.94 ± 10.52	<.0001
Glucose, mg/dL	97.01 ± 22.08	97.76 ± 22.16	0.0103
Total cholesterol, mg/dL	200.77 ± 42.77	201.87 ± 44.1	0.0547
HDL-C, mg/dL	59.08 ± 36.48	58.16 ± 35.56	0.0601
LDL-C, mg/dL	121.44 ± 73.35	124.55 ± 146.42	0.0016
TG, mg/dL	103.54 (103.48–103.61)	106.7 (105.23–108.19)	<.0001

^a^Low income was defined as a lower quintile of yearly income (lower 20%). ^b^Alcohol consumption: mild (<30 g of alcohol a day), heavy (≥30 g of alcohol a day). ^c^Strenuous exercise done for at least 20 minutes 1–4 times/week. Presented as n (%), mean ± SD, or median (IQR). BMI, body mass index; DBP, diastolic blood pressure; HDL-C, high-density lipoprotein cholesterol; IQR, interquartile range; LDL-C, low-density lipoprotein cholesterol; SBP, systolic blood pressure; SD, standard deviation; TG, triglycerides; WC, waist circumference.

### Definition of Metabolic Syndrome

In this study, metabolic syndrome was described according to the criteria of the National Cholesterol Education Program Adult Treatment Panel III (29). In detail, metabolic syndrome was diagnosed when a person met at least three of the following five components: (I) Women with a WC of ≥85cm, suggesting abdominal obesity according to the definition from the Korean Society for the Study of Obesity (30); (II) SBP ≥ 130 mmHg or DBP ≥ 85 mmHg, or who have been prescribed antihypertensive drugs; (III) serum TG ≥ 150 mg/dL or who have been prescribed lipid-lowering drugs; (IV) serum HDL-C <50 mg/dL or who have been prescribed lipid-lowering drugs; (V) Fasting blood glucose ≥ 100 mg/dL or those who have been used hypoglycemic drugs.

### Statistical Analysis

Differences in baseline characteristics were evaluated between the groups with metabolic syndrome versus those without metabolic syndrome using Student’s t-test for continuous variables and Pearson’s chi-square test for categorical variables. Endometrial cancer incidence rates were calculated by dividing the number of incident cases by 1000 person-years. The hazard ratios (HRs) and 95% confidence intervals (CIs) were calculated using multivariate Cox proportional hazards regression models, and these models were used for exploring the correlation between metabolic syndrome and its components and the occurrence of endometrial cancer. We analyzed the effect of metabolic syndrome on the development of endometrial cancer according to menopause status. Also, we conducted subgroup analyses to determine how the role of metabolic syndrome on the incidence of endometrial cancer differed by the menopause status. In multivariate analyses, age, sex, smoking status (3 levels), alcohol consumption (3 levels), and regular physical activity were adjusted. All statistical analyses were conducted using R statistical software (version 3.4.4; R Foundation for Statistical Computing, Vienna, Austria; http://www.R-project.org) and SAS statistical software (version 9.4; SAS Institute, Cary, NC, USA). A *p*-values <0.05 was considered as statistically significant.

## Results

### Baseline Characteristics of the Study Population

Among 2,824,107 women, 5,604 were diagnosed with endometrial cancer, while 2,818,503 were not (control group). The incidence rate of endometrial cancer during the year 2009 was 0.24 per 1,000 person-years. **Table 1** showed the baseline characteristics of the study population. Women in the endometrial cancer group was significantly younger than those in the control group (mean, 53.28 *vs*. 54.03 years; *p* <.0001). The endometrial cancer group had a lower trend of HDL-C levels than that in the control group, but without statistical significance (*p* =0.0601). However, the endometrial cancer group was more likely to have hypertension and dyslipidemia than the control group. The endometrial cancer group had significantly higher BMI (mean, 24.54 *vs*. 23.72 kg/m^2^; *p* <.0001) and WC (mean, 79.46 *vs*. 77.89 cm; *p* <.0001), compared to the control group.

### Metabolic Syndrome and Its Components Increase the Risk of Incidence of Endometrial Cancer According to the Menopausal Status

**Figure 2:**
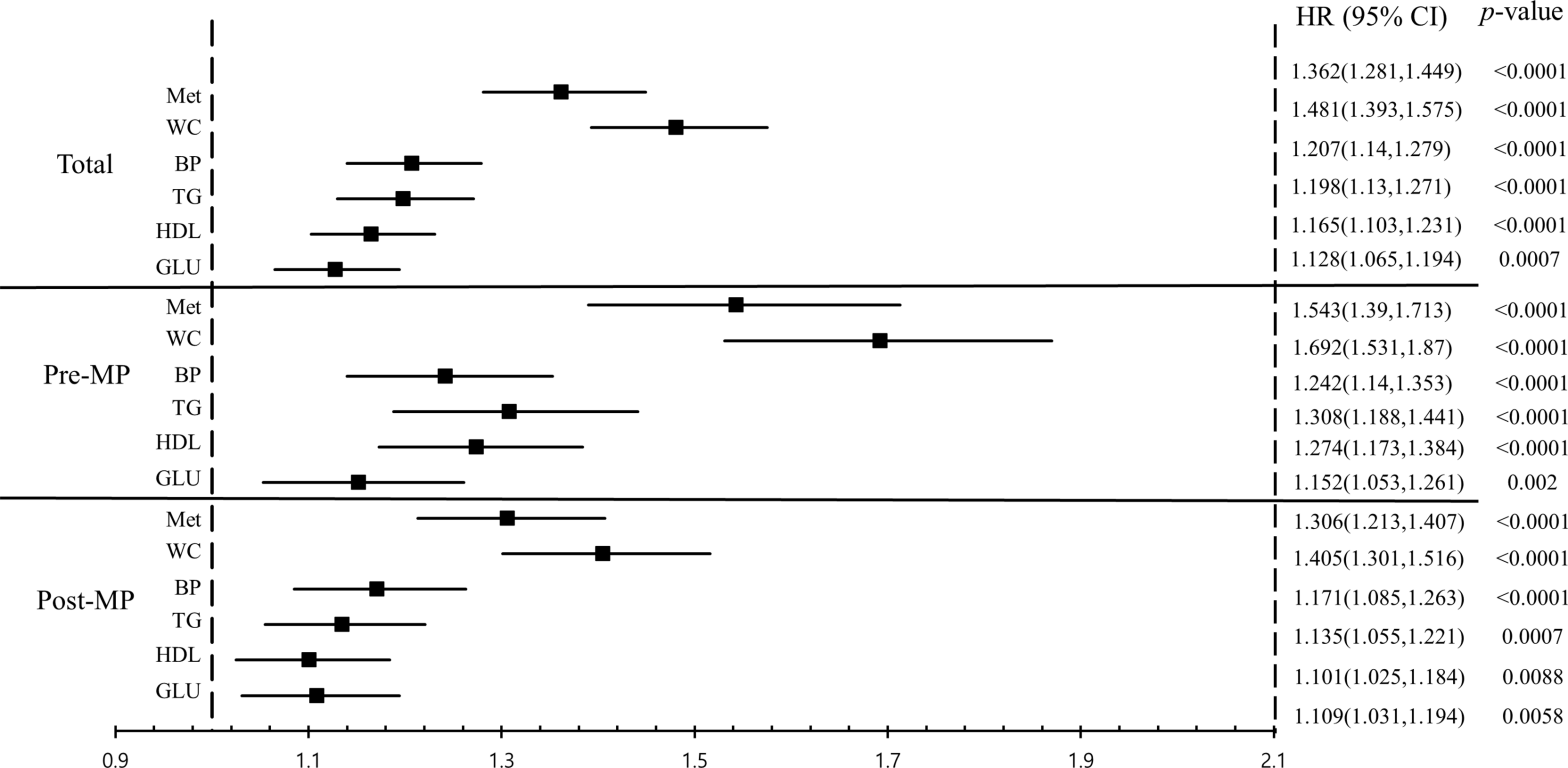
Association of metabolic syndrome and its components and incidence of endometrial cancer among all women, pre-menopausal women, and post-menopausal women. Age, sex, smoking, alcohol consumption, and regular exercise were adjusted. BP, blood pressure; CI, confidence interval; GLU, fasting blood glucose; HDL-C, high-density lipoprotein cholesterol; HR, hazard ratio; Met, metabolic syndrome; Post-MP, post-menopausal; Pre-MP, pre-menopausal; TG, triglycerides; WC, waist circumference.

Of the study population, 736,846 women had metabolic syndrome, whereas 2,087,261 women did not. In the metabolic syndrome group, 1,703 were diagnosed with endometrial cancer, while 3,901 were diagnosed with endometrial cancer in the non-metabolic syndrome group. The incidence rate of endometrial cancer in the metabolic syndrome group was higher than that in the non-metabolic syndrome group (0.28 *vs*. 0.23 per 1,000 person-years; *p* <.0001). The results of the incidence rate of endometrial cancer according to the metabolic syndrome and its components are shown in **Table S1**. In multivariate analysis, the presence of metabolic syndrome was associated with an increased risk of endometrial cancer (HR, 1.362; 95% CI, 1.281–1.449) (**Figure 2**).

We also investigated the association between the risk of endometrial cancer and each component of metabolic syndrome (WC, BP, serum TG and HDL-C levels, and fasting blood glucose). All five components of metabolic syndrome were associated with an increased risk of developing endometrial cancer. Among the five components, abdominal obesity showed the highest risk for developing endometrial cancer (HR, 1.481; 95% CI, 1.393–1.575) (**Figure 2**).

Furthermore, we examined the effect of metabolic syndrome on the incidence of endometrial cancer according to the menopausal status (**Figure 2**). There was a significant interaction between menopausal status and metabolic syndrome on the risk of endometrial cancer (*p* for interaction <.0001). The incidence rate of endometrial cancer in the pre-menopausal women with the metabolic syndrome was approximately 1.7 times higher than that in the post-menopausal women with the metabolic syndrome group (0.42 *vs*. 0.24 per 1,000 person-years; *p* <.0001) (**Table S1**). For the women with metabolic syndrome, the risk of developing endometrial cancer incidence was higher in the pre-menopausal subgroup (HR, 1.543; 95% CI, 1.39–1.713) compared to that in the post-menopausal subgroup (HR, 1.306; 95% CI, 1.213–1.407).

**Figure 2** also showed five components of metabolic syndrome were related to the increased risk of endometrial cancer in both pre-menopausal and post-menopausal subgroups. In the pre-menopausal subgroup, abdominal obesity (HR, 1.692; 95% CI, 1.531–1.87), BP (HR, 1.242; 95% CI, 1.14–1.353), TG (HR, 1.308; 95% CI, 1.188–1.441), HDL-C (HR, 1.274; 95% CI, 1.173–1.384), and fasting blood glucose (HR, 1.152; 95% CI, 1.053–1.261) were significantly associated with the increased risk of endometrial cancer. In addition, each component of metabolic syndrome was associated with the incidence of endometrial cancer in the post-menopausal subgroup, abdominal obesity (HR, 1.405; 95% CI, 1.301–1.516), BP (HR, 1.171; 95% CI, 1.085–1.263), TG (HR, 1.135; 95% CI, 1.055–1.221), HDL-C (HR, 1.101; 95% CI, 1.025–1.184), and fasting blood glucose (HR, 1.109; 95% CI, 1.031–1.194). Among the five components of metabolic syndrome, abdominal obesity was the most important factor to develop endometrial cancer, more evident in pre-menopausal women than in post-menopausal women (*p* for interaction <.0001). Similarly, five components of metabolic syndrome were more closely associated with the incidence of endometrial cancer in the pre-menopausal subgroup than in the post-menopausal subgroup (for WC, PB, TG and HDL-C, all *p* for interaction <.0001 respectively, and for fasting blood glucose, *p* for interaction 0.0188).

### The Number of Metabolic Syndrome Components and Risk of Endometrial Cancer According to the Menopausal Status

**Figure 3:**
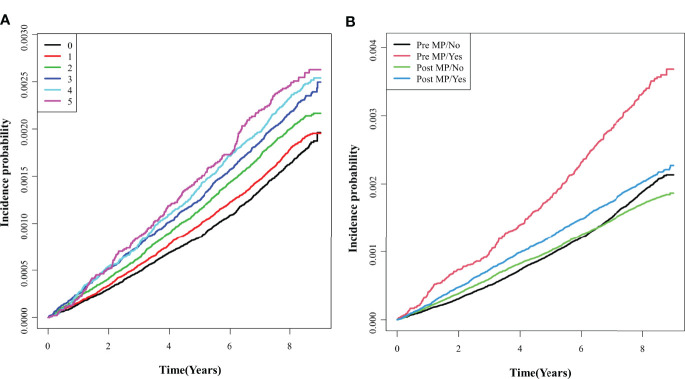
Comparisons of endometrial cancer incidence according to the number of metabolic syndrome components. **(A)** Total group; **(B)** Pre- and post- menopausal subgroups. Pre MP/No, pre-menopausal women without the metabolic syndrome; Pre MP/Yes, pre-menopausal women with the metabolic syndrome; Post MP/No, post-menopausal women without the metabolic syndrome; Post MP/Yes, post-menopausal women with the metabolic syndrome.

We showed the longitudinal associations between the number of metabolic syndrome components and endometrial cancer incidence probability using the cumulative incidence competing for risk methods (**Figure 3**). During 8.37 years of the median follow-up period, Kaplan-Meier curves showed the incidence probabilities of endometrial cancer according to the number (0–5) of components of the metabolic syndrome in the pre-menopausal and post-menopausal subgroups. The incidence of endometrial cancer increased significantly as the number of metabolic syndrome components increased (log-rank *p* <.0001) in all study population (**Figure 3A**). **Figure 3B** shows the pre-menopausal subgroup tended to have an rapid increase in the incidence of endometrial cancer when they had metabolic syndrome (≥3 metabolic syndrome components), than the post-menopausal subgroup (*p* for interaction <.0001).

**Figure 4:**
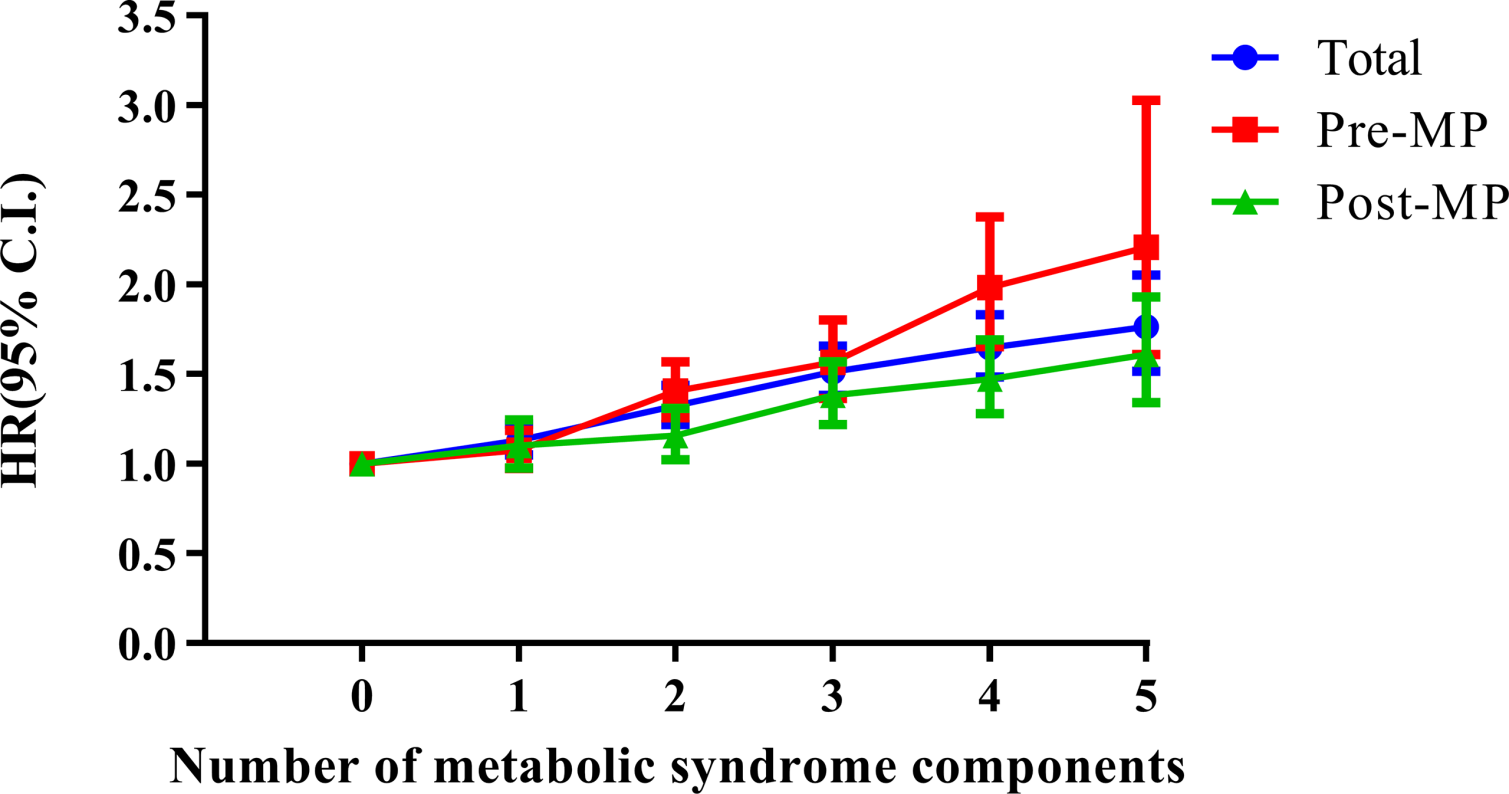
Combined effects of metabolic syndrome and menopausal status on the risk of endometrial cancer. Age, sex, smoking, alcohol consumption, and regular exercise were adjusted. Pre-MP, pre-menopausal; Post-MP, post-menopausal.

Next, we conducted multivariate analyses (**Figure 4** and **Table 2**) and found that women with three components of the metabolic syndrome having at an increased risk of developing endometrial cancer than those who did not have any metabolic syndrome component in total groups (HR, 1.511; 95% CI, 1.381–1.654) (**Table 2**). In both pre-menopausal and post-menopausal subgroups, the incidence rate of endometrial cancer gradually increased with each addition of one component of metabolic syndrome. Interestingly, with the addition of abdominal obesity, the incidence of endometrial cancer dramatically increased compared to the addition of other components of metabolic syndrome in both pre-menopausal and post-menopausal subgroups (**Table S2**). The pre-menopausal subgroup with all five metabolic syndrome components had a 2.2 times higher risk of developing endometrial cancer compared with those who did not have any metabolic syndrome (HR, 2.197; 95% CI, 1.602–3.015), while the post-menopausal subgroup with all five metabolic syndrome components had a 1.6 times higher risk (HR, 1.619; 95% CI, 1.348–1.945) than those who did not have any metabolic syndrome components.

**Table 2:** Incidence of endometrial cancer according to the number of metabolic syndrome components among all women, pre-menopausal women, and post-menopausal women.

Number of metabolic syndrome components	Events	Person-years	Incidence rate	Adjusted HR^a^ (95% CI)
***Total***				
0	1247	6,110,523.53	0.20849	1 (Ref.)
1	1419	6,335,084.2	0.22399	1.131 (1.048–1.221)
2	1208	4,854,526.22	0.24884	1.323 (1.219–1.436)
3	928	3,398,326.93	0.27308	1.511 (1.381–1.654)
4	566	1,953,874.2	0.28968	1.647 (1.482–1.831)
5	209	689,186.49	0.30326	1.763 (1.515–2.053)
***Pre- menopausal***				
0	857	4,088,049.06	0.20964	1 (Ref.)
1	736	3,122,563.25	0.2357	1.074 (0.973–1.186)
2	513	1,593,082.9	0.32202	1.404 (1.257–1.568)
3	270	722,398.12	0.37376	1.566 (1.363–1.8)
4	140	285,235.5	0.49082	1.982 (1.653–2.377)
5	41	72,431.73	0.56605	2.205 (1.608–3.025)
***Post-menopausal***				
0	417	2,022,474.47	0.20618	1 (Ref.)
1	683	3,212,520.95	0.21261	1.101 (0.975–1.245)
2	695	3,261,443.33	0.2131	1.157 (1.023–1.309)
3	658	2,675,928.81	0.2459	1.382 (1.218–1.567)
4	426	1,668,638.7	0.2533	1.471 (1.28–1.691)
5	168	616,754.75	0.27239	1.608 (1.339–1.93)

CI, confidence interval; HR, hazard ratio; Ref., reference. ^a^Adjusted for age, sex, smoking, alcohol consumption, regular exercise; HR, hazard ratio; CI, 95% confidence interval.

## Discussion

To our knowledge, this nationwide population-based cohort study was the first evaluating the risk of developing endometrial cancer in women with metabolic syndrome or each metabolic syndrome component according to the menopausal status. In this study, endometrial cancer risk was considerably higher in individuals who had metabolic syndrome components than those who did not. The impact of metabolic syndrome on the risk of endometrial cancer varied depending on the menopausal status: the risk of endometrial cancer from metabolic syndrome was higher in the pre-menopausal subgroup than post-menopausal subgroup.

Endometrial cancer predominantly occurs in post-menopause women. Moreover, the incidence of endometrial cancer is increasing in most countries, mainly due to the increasing incidence in pre-menopausal women. While the incidence of endometrial cancer among pre-menopausal women has decreased in European nations, including Denmark, the Czech Republic, and the Netherlands, it has gradually increased in Asian nations, including China, Japan, and Singapore (1). Especially, in Korea, the incidence of endometrial cancer between 1999 and 2017 increased most rapidly in the young women under 30 years and 30–39 years age group (annual percent change (APC), 8.7% and 7.4%, respectively). On the other hand, the 40–49 years and 50–59 years age groups, showed relatively slow increase during the same period (APC, 5.1% and 5.7% respectively) (6). Similarly, the incidence rate of endometrial cancer has increased most rapidly in women under 50 years in New Zealand (31). Nonetheless, these studies have limitations on comprehensively demonstrating the reasons why pre-menopausal women have a higher risk of endometrial cancer. Guo et al. reported that the significantly increased incidence of endometrial cancer in the 20–39 years age group was related with the concurrent increasing prevalence of obesity in young women in the United States (32). However, this study used only a group of pre-menopausal women and did not confirm the association between obesity-induced metabolic syndrome and endometrial cancer. Previous researchers reported positive associations between metabolic syndrome and obesity and the incidence of female cancers, including endometrial cancer (33–35). As the prevalence of metabolic syndrome has markedly increased affecting one quarter of the global population (10), the prevalence of endometrial cancer is predicted to further rise in the future.

The positive correlation between metabolic syndrome and the risk of endometrial cancer in our study is supported by previous studies. An Italian case-control study including 454 endometrial cancer cases and 798 controls showed a significant association between metabolic syndrome and risk of endometrial cancer. Among components of metabolic syndrome, obesity appeared to be a critical factor for developing endometrial cancer (23). Another case-control study in the United States investigated the effect of metabolic syndrome on the incidence of endometrial cancer using the large population data from 1993 to 2007 (16,323 cases of endometrial cancer and 100,751 controls) which included women aged 65 and older. Among patients diagnosed with metabolic syndrome, the risk of endometrial cancer was doubled in obese patients compared to that of non-obese patients (36). Although only post-menopausal women were included, Rhonda et al. found that endometrial cancer risk was doubled in women who had metabolic syndrome and WC > 88cm (21). To date, most previous studies did not examine the relationship between metabolic syndrome and endometrial cancer according to the menopausal status, and they only discussed the relationship in post-menopausal women. In addition, the association between metabolic syndrome and endometrial cancer in recent studies is limited to Western women (37). Considering that incidence and mortality rates of endometrial cancer have ethnic and geographic variations (38), large cohort studies are also required in Asian women. Therefore, we performed a comprehensive national population-based cohort study of Korean females.

We showed that there was a higher incidence of endometrial cancer in pre-menopausal women with metabolic syndrome than post-menopausal counterparts. Similarly, Friedenreich et al. found that metabolic syndrome was highly linked to the occurrence of endometrial cancer and the risk of endometrial cancer was higher in pre-menopausal women than in post-menopausal women in the presence of the metabolic syndrome (22). Furthermore, we discovered that abdominal obesity was a major determinant in the development of endometrial cancer among the components of metabolic syndrome. Pre-menopausal women were shown to be 69% more likely to develop endometrial cancer when they have a higher WC (≥85cm), while post-menopausal women with a WC of ≥85cm had a 40% increased risk. When pre-menopausal women have abdominal obesity, their risk of endometrial cancer rises considerably. Recently, our research team reported on the female-specific cancer risk according to obesity and menopausal status using a nationwide cohort in Korea. In the pre-menopausal women, a WC of <75cm was related with a protective effect against the risk of endometrial cancer, but a WC of ≥95cm was substantially linked with an elevated risk of endometrial cancer (39). Soliman et al. have shown that most patients diagnosed with endometrial cancer at a young age were obese (40). Considering that estrogen is a well-known endometrial growth factor, excessive estrogen produced by redundant adipocytes of obese seems to be responsible for continuous stimulation of the endometrium, endometrial hyperplasia and endometrial cancer (41, 42).

Other studies have reported that metabolic syndrome components other than WC are not associated with the development of endometrial cancer (21). However, our findings suggested that other metabolic syndrome components (BP, TG, HDL-C and fasting blood glucose) could be additional risk factors for developing endometrial cancer. For example, high BP increased the risk of endometrial cancer by 20% in the total population. Like WC, pre-menopausal women with high BP also showed a higher risk than post-menopausal counterparts. Other metabolic syndrome components also significantly increased the incidence of endometrial cancer, suggesting that it might potentially be a useful criterion for predicting the risk of endometrial cancer. This finding is important because metabolic syndrome and obesity can be managed and prevented with exercise and diet control (43).

Our study has several limitations. First, we only used Korean data and ethnicity differences were not discussed here. Second, we did not differentiate the histologic subtypes or stages of endometrial cancer due to the lack of accessibility to the specific clinicopathologic data. Third, dietary habits and lifestyles that could potentially modulate the metabolic syndrome were not included. Fourth, smoking data were not detailed in the analysis because the data we used did not include the duration of smoking and the cessation data. Fifth, we did not analyze the duration of the metabolic syndrome, which may also affect the development of endometrial cancer. Finally, as this study is a large-scale retrospective study, the differences in health examinations and laboratory measurements (etc., different assays for blood tests, time of fasting for those blood tests, how WC was measured) performed to determine metabolic syndrome should be considered. Nevertheless, this study covered more than 2.8 million South Korean women and had a long follow-up period (median, 8.37 years), providing sufficient time for monitoring endometrial cancer development. Also, we eliminated individuals with less than a year of follow-up to establish a causal link and minimize detection bias. To the best of our knowledge, this is the largest cohort to examine the effects of metabolic syndrome on the development of endometrial cancer according to menopause status.

## Conclusion

In conclusion, this large, nationwide population cohort study provides evidence of the effect of metabolic syndrome on the development of endometrial cancer according to the menopausal status. To summarize our findings, women with metabolic syndrome, especially pre-menopausal women with abdominal obesity, were at high risk of developing endometrial cancer. For pre-menopausal women as well as post-menopausal women with metabolic syndrome, frequent endometrial cancer surveillance and risk modulation by supervised exercise in combination with nutrition programs might be necessary.

## Data Availability Statement

The datasets presented in this article are not readily available because according to Korean law, we are not allowed to transfer any data files to a third party. However, data are available from the Korea National Health Insurance Sharing Service Institutional Data Access/Ethics Committee (https://nhiss.nhis.or.kr/bd/ay/bdaya001iv.do) for researchers who meet the criteria for access to confidential data. Researchers can apply for the National Health Insurance data sharing service upon approval of the institutional review board of their institution. Requests to access the datasets should be directed to Korea National Health Insurance Sharing Service Institutional Data Access/Ethics Committee (https://nhiss.nhis.or.kr/bd/ay/bdaya001iv.do).

## Ethics Statement

The studies involving human participants were reviewed and approved by The Institutional Review Board of Seoul National University Hospital (No. 1811-048-983). Written informed consent for participation was not required for this study in accordance with the national legislation and the institutional requirements.

## Author Contributions

HJ contributed to the study design, data interpretation, and writing of the first draft of the manuscript. SK contributed to data interpretation and review of the manuscript. WW, AS, YH, JK, IP, and JL were involved in data interpretation. JY was involved in data analysis. K-DH contributed to the study design, analysis of the data, and advice on the study conception. YS supervised the entire project. All authors reviewed or revised the manuscript and approved the final manuscript for submission.

## Funding

This work was supported by BK21 Plus Program of the Department of Agricultural Biotechnology, Seoul National University (Seoul, Korea).

## Conflict of Interest

Author YH was employed by the company SK Biopharmaceuticals Co., Ltd.

The remaining authors declare that the research was conducted in the absence of any commercial or financial relationships that could be construed as a potential conflict of interest.

## Publisher’s Note

All claims expressed in this article are solely those of the authors and do not necessarily represent those of their affiliated organizations, or those of the publisher, the editors and the reviewers. Any product that may be evaluated in this article, or claim that may be made by its manufacturer, is not guaranteed or endorsed by the publisher.
