# Supplementary material for: Metabolic Syndrome as a Risk Factor of Endometrial Cancer: A Nationwide Population-Based Cohort Study of 2.8 Million Women in South Korea
Source: Front Oncol. 2022 Jun 16;12:872995. doi: 10.3389/fonc.2022.872995 (PMC9243219; doi:10.3389/fonc.2022.872995)
Supplement: Supplementary file 1 [file Table_1.docx]

Supplementary Material

**Table S1.** The incidence rate of endometrial cancer according to the metabolic syndrome and its components among all women, pre-menopausal women, and post-menopausal women

| **Met or component** | **N** | **Events** | **Person-years** | **Incidence rate^a^** |
| --- | --- | --- | --- | --- |
| **Total** | | | | |
| **Met** |  |  |  |  |
| No | 2087261 | 3901 | 17300133.96 | 0.22549 |
| Yes | 736846 | 1703 | 6041387.61 | 0.28189 |
| **WC (cm)** |  |  |  |  |
| <85 | 2228589 | 4092 | 18442573.02 | 0.22188 |
| ≥85 | 595518 | 1512 | 4898948.56 | 0.30864 |
| **BP** |  |  |  |  |
| Normal | 1551005 | 2941 | 12890744.95 | 0.22815 |
| High^b^ | 1273102 | 2663 | 10450776.63 | 0.25481 |
| **TG (mg/dL)** |  |  |  |  |
| Low (< 150) | 2023012 | 3863 | 16739658.12 | 0.23077 |
| High (≥ 150) | 801095 | 1741 | 6601863.45 | 0.26371 |
| **HDL-C (mg/dL)** |  |  |  |  |
| High (≥50) | 1795956 | 3397 | 14857087.44 | 0.22865 |
| Low (<50) | 1028151 | 2207 | 8484434.13 | 0.26012 |
| **GLU (mg/dL)** |  |  |  |  |
| Normal (< 100) | 1963136 | 3799 | 16276997.68 | 0.2334 |
| High (≥ 100) | 860971 | 1805 | 7064523.89 | 0.2555 |
| **Pre- MP** | | | | |
| **Met** |  |  |  |  |
| No | 1059580 | 2106 | 8803695.21 | 0.23922 |
| Yes | 130098 | 451 | 1080065.35 | 0.41757 |
| **WC (cm)** |  |  |  |  |
| <85 | 1054578 | 2074 | 8762080.23 | 0.2367 |
| ≥85 | 135100 | 483 | 1121680.33 | 0.4306 |
| **BP** |  |  |  |  |
| Normal | 888763 | 1734 | 7387103.65 | 0.23473 |
| High^b^ | 300915 | 823 | 2496656.92 | 0.32964 |
| **TG (mg/dL)** |  |  |  |  |
| Low (< 150) | 1004309 | 2022 | 8343304.5 | 0.24235 |
| High (≥ 150) | 185369 | 535 | 1540456.06 | 0.3473 |
| **HDL-C (mg/dL)** |  |  |  |  |
| High (≥50) | 866939 | 1705 | 7196750.17 | 0.23691 |
| Low (<50) | 322739 | 852 | 2687010.4 | 0.31708 |
| **GLU (mg/dL)** |  |  |  |  |
| Normal (< 100) | 932492 | 1913 | 7750540.21 | 0.24682 |
| High (≥ 100) | 257186 | 644 | 2133220.36 | 0.30189 |
| **Post-MP** | | | | |
| **Met** |  |  |  |  |
| No | 1027681 | 1795 | 8496438.75 | 0.21126 |
| Yes | 606748 | 1252 | 4961322.26 | 0.24235 |
| **WC (cm)** |  |  |  |  |
| <85 | 1174011 | 2018 | 9680492.78 | 0.20846 |
| ≥85 | 460418 | 1029 | 3777268.22 | 0.27242 |
| **BP** |  |  |  |  |
| Normal | 662242 | 1207 | 5503641.3 | 0.21931 |
| High^b^ | 972187 | 1840 | 7954119.71 | 0.23133 |
| **TG (mg/dL)** |  |  |  |  |
| Low (< 150) | 1018703 | 1841 | 8396353.61 | 0.21926 |
| High (≥ 150) | 615726 | 1206 | 5061407.39 | 0.23827 |
| **HDL-C (mg/dL)** |  |  |  |  |
| High (≥50) | 929017 | 1692 | 7660337.27 | 0.22088 |
| Low (<50) | 705412 | 1355 | 5797423.74 | 0.23372 |
| **GLU (mg/dL)** |  |  |  |  |
| Normal (< 100) | 1030644 | 1886 | 8526457.47 | 0.22119 |
| High (≥ 100) | 603785 | 1161 | 4931303.53 | 0.23543 |

^a^Endometrial cancer incidence per 1,000 person-years.

^b^High BP, systolic BP ≥130 and/or diastolic BP ≥85 mmHg.

Abbreviations: BP, blood pressure; GLU, fasting blood glucose; HDL-C, high-density lipoprotein cholesterol; Met, metabolic syndrome; Post-MP, post-menopausal; Pre-MP, pre-menopausal; TG, triglycerides; WC, waist circumference.

**Table S2.** Incidence of endometrial cancer according to the addition of metabolic syndrome components among all women, pre-menopausal women, and post-menopausal women

| **WC** | **BP** | **TG** | **HDL-C** | **GLU** | **N** | **Events** | **Person-years** | **Incidence rate^a^** | **Adjusted HR (95 %CI)^b^** |
| --- | --- | --- | --- | --- | --- | --- | --- | --- | --- |
| **TOTAL** | | | | | | | | | |
| No | No | No | No | No | 735130 | 1274 | 6110523.53 | 0.20849 | 1(ref.) |
| No | No | No | No | Yes | 159297 | 246 | 1320461.44 | 0.1863 | 0.922(0.804,1.057) |
| No | No | No | Yes | No | 197139 | 387 | 1642034.91 | 0.23568 | 1.144(1.021,1.282) |
| No | No | No | Yes | Yes | 47568 | 88 | 394216.68 | 0.22323 | 1.116(0.899,1.386) |
| No | No | Yes | No | No | 62656 | 124 | 520910.54 | 0.23804 | 1.213(1.008,1.459) |
| No | No | Yes | No | Yes | 25325 | 54 | 209554.32 | 0.25769 | 1.346(1.025,1.768) |
| No | No | Yes | Yes | No | 93811 | 196 | 782400.84 | 0.25051 | 1.296(1.114,1.508) |
| No | No | Yes | Yes | Yes | 44551 | 84 | 369650.87 | 0.22724 | 1.201(0.962,1.5) |
| No | Yes | No | No | No | 286338 | 519 | 2364201.82 | 0.21952 | 1.156(1.041,1.282) |
| No | Yes | No | No | Yes | 129158 | 255 | 1057312.83 | 0.24118 | 1.311(1.143,1.504) |
| No | Yes | No | Yes | No | 96106 | 183 | 790785.86 | 0.23142 | 1.25(1.068,1.462) |
| No | Yes | No | Yes | Yes | 48504 | 98 | 393072.47 | 0.24932 | 1.389(1.128,1.711) |
| No | Yes | Yes | No | No | 55496 | 83 | 457277.95 | 0.18151 | 1.004(0.802,1.256) |
| No | Yes | Yes | No | Yes | 39485 | 85 | 321314.51 | 0.26454 | 1.495(1.197,1.866) |
| No | Yes | Yes | Yes | No | 112326 | 230 | 927882.01 | 0.24788 | 1.389(1.202,1.605) |
| No | Yes | Yes | Yes | Yes | 95699 | 186 | 780972.42 | 0.23816 | 1.358(1.159,1.592) |
| Yes | No | No | No | No | 58650 | 143 | 487475.5 | 0.29335 | 1.496(1.258,1.78) |
| Yes | No | No | No | Yes | 25119 | 73 | 207954.49 | 0.35104 | 1.835(1.449,2.325) |
| Yes | No | No | Yes | No | 25689 | 64 | 214090.3 | 0.29894 | 1.529(1.189,1.966) |
| Yes | No | No | Yes | Yes | 11421 | 29 | 94553.75 | 0.3067 | 1.611(1.114,2.329) |
| Yes | No | Yes | No | No | 13797 | 34 | 114700.57 | 0.29642 | 1.566(1.113,2.202) |
| Yes | No | Yes | No | Yes | 9033 | 23 | 74710.24 | 0.30786 | 1.656(1.096,2.503) |
| Yes | No | Yes | Yes | No | 23808 | 69 | 198424.51 | 0.34774 | 1.859(1.458,2.372) |
| Yes | No | Yes | Yes | Yes | 18011 | 53 | 149082.45 | 0.35551 | 1.933(1.467,2.548) |
| Yes | Yes | No | No | No | 75982 | 178 | 626232.37 | 0.28424 | 1.586(1.351,1.861) |
| Yes | Yes | No | No | Yes | 61172 | 161 | 499222.01 | 0.3225 | 1.844(1.558,2.181) |
| Yes | Yes | No | Yes | No | 35302 | 77 | 290502.86 | 0.26506 | 1.498(1.187,1.89) |
| Yes | Yes | No | Yes | Yes | 30437 | 88 | 247017.29 | 0.35625 | 2.057(1.651,2.562) |
| Yes | Yes | Yes | No | No | 27813 | 72 | 228993.7 | 0.31442 | 1.798(1.414,2.286) |
| Yes | Yes | Yes | No | Yes | 31505 | 73 | 256241.61 | 0.28489 | 1.651(1.3,2.097) |
| Yes | Yes | Yes | Yes | No | 63093 | 166 | 520560.42 | 0.31889 | 1.844(1.561,2.178) |
| Yes | Yes | Yes | Yes | Yes | 84686 | 209 | 689186.49 | 0.30326 | 1.776(1.525,2.069) |
| **Pre- MP** | | | | | | | | | |
| No | No | No | No | No | 492123 | 857 | 4088049.06 | 0.20964 | 1(ref.) |
| No | No | No | No | Yes | 90612 | 150 | 751713.53 | 0.19954 | 0.924(0.777,1.1) |
| No | No | No | Yes | No | 122666 | 245 | 1022283.93 | 0.23966 | 1.126(0.976,1.298) |
| No | No | No | Yes | Yes | 25050 | 54 | 208536.62 | 0.25895 | 1.182(0.898,1.556) |
| No | No | Yes | No | No | 28911 | 53 | 240290.94 | 0.22057 | 1.01(0.765,1.334) |
| No | No | Yes | No | Yes | 10145 | 24 | 84111.84 | 0.28533 | 1.29(0.859,1.936) |
| No | No | Yes | Yes | No | 34451 | 96 | 287159.42 | 0.33431 | 1.473(1.192,1.82) |
| No | No | Yes | Yes | Yes | 13768 | 34 | 114397.81 | 0.29721 | 1.291(0.916,1.819) |
| No | Yes | No | No | No | 107086 | 216 | 888438.06 | 0.24312 | 1.054(0.907,1.225) |
| No | Yes | No | No | Yes | 36871 | 110 | 305098.32 | 0.36054 | 1.525(1.248,1.862) |
| No | Yes | No | Yes | No | 29098 | 74 | 242210.82 | 0.30552 | 1.298(1.023,1.648) |
| No | Yes | No | Yes | Yes | 10837 | 27 | 89934.79 | 0.30022 | 1.247(0.849,1.83) |
| No | Yes | Yes | No | No | 13513 | 24 | 112057.4 | 0.21418 | 0.901(0.6,1.353) |
| No | Yes | Yes | No | Yes | 7816 | 21 | 64569.58 | 0.32523 | 1.352(0.876,2.086) |
| No | Yes | Yes | Yes | No | 18825 | 49 | 156810.96 | 0.31248 | 1.247(0.933,1.666) |
| No | Yes | Yes | Yes | Yes | 12806 | 40 | 106417.16 | 0.37588 | 1.467(1.066,2.018) |
| Yes | No | No | No | No | 26466 | 72 | 219836.8 | 0.32752 | 1.496(1.176,1.903) |
| Yes | No | No | No | Yes | 9569 | 35 | 79356.36 | 0.44105 | 1.978(1.41,2.774) |
| Yes | No | No | Yes | No | 11514 | 34 | 96025.39 | 0.35407 | 1.62(1.15,2.283) |
| Yes | No | No | Yes | Yes | 4309 | 8 | 35853.55 | 0.22313 | 1.004(0.5,2.014) |
| Yes | No | Yes | No | No | 4825 | 12 | 40096.27 | 0.29928 | 1.374(0.777,2.43) |
| Yes | No | Yes | No | Yes | 2759 | 11 | 22891.96 | 0.48052 | 2.163(1.193,3.922) |
| Yes | No | Yes | Yes | No | 7025 | 33 | 58521.79 | 0.56389 | 2.493(1.76,3.531) |
| Yes | No | Yes | Yes | Yes | 4570 | 16 | 37978.39 | 0.42129 | 1.861(1.134,3.052) |
| Yes | Yes | No | No | No | 16680 | 50 | 138430.46 | 0.36119 | 1.514(1.137,2.017) |
| Yes | Yes | No | No | Yes | 10104 | 36 | 83566.35 | 0.4308 | 1.758(1.258,2.458) |
| Yes | Yes | No | Yes | No | 6784 | 30 | 56366.19 | 0.53223 | 2.227(1.546,3.208) |
| Yes | Yes | No | Yes | Yes | 4540 | 24 | 37604.27 | 0.63823 | 2.622(1.746,3.938) |
| Yes | Yes | Yes | No | No | 4771 | 21 | 39485.13 | 0.53185 | 2.212(1.433,3.415) |
| Yes | Yes | Yes | No | Yes | 4688 | 13 | 38758.1 | 0.33541 | 1.382(0.798,2.393) |
| Yes | Yes | Yes | Yes | No | 7754 | 47 | 64477.59 | 0.72894 | 2.88(2.142,3.873) |
| Yes | Yes | Yes | Yes | Yes | 8742 | 41 | 72431.73 | 0.56605 | 2.197(1.602,3.015) |
| **Post-MP** | | | | | | | | | |
| No | No | No | No | No | 243007 | 417 | 2022474.47 | 0.20618 | 1(ref.) |
| No | No | No | No | Yes | 68685 | 96 | 568747.92 | 0.16879 | 0.844(0.676,1.054) |
| No | No | No | Yes | No | 74473 | 142 | 619750.98 | 0.22912 | 1.151(0.951,1.393) |
| No | No | No | Yes | Yes | 22518 | 34 | 185680.06 | 0.18311 | 0.947(0.668,1.344) |
| No | No | Yes | No | No | 33745 | 71 | 280619.6 | 0.25301 | 1.276(0.992,1.641) |
| No | No | Yes | No | Yes | 15180 | 30 | 125442.49 | 0.23915 | 1.231(0.849,1.783) |
| No | No | Yes | Yes | No | 59360 | 100 | 495241.42 | 0.20192 | 1.035(0.831,1.287) |
| No | No | Yes | Yes | Yes | 30783 | 50 | 255253.06 | 0.19588 | 1.025(0.764,1.375) |
| No | Yes | No | No | No | 179252 | 303 | 1475763.75 | 0.20532 | 1.105(0.952,1.283) |
| No | Yes | No | No | Yes | 92287 | 145 | 752214.51 | 0.19276 | 1.071(0.885,1.295) |
| No | Yes | No | Yes | No | 67008 | 109 | 548575.04 | 0.1987 | 1.114(0.901,1.378) |
| No | Yes | No | Yes | Yes | 37667 | 71 | 303137.68 | 0.23422 | 1.352(1.049,1.743) |
| No | Yes | Yes | No | No | 41983 | 59 | 345220.55 | 0.17091 | 0.954(0.725,1.254) |
| No | Yes | Yes | No | Yes | 31669 | 64 | 256744.93 | 0.24927 | 1.419(1.089,1.85) |
| No | Yes | Yes | Yes | No | 93501 | 181 | 771071.05 | 0.23474 | 1.321(1.107,1.576) |
| No | Yes | Yes | Yes | Yes | 82893 | 146 | 674555.26 | 0.21644 | 1.241(1.025,1.502) |
| Yes | No | No | No | No | 32184 | 71 | 267638.69 | 0.26528 | 1.375(1.069,1.769) |
| Yes | No | No | No | Yes | 15550 | 38 | 128598.13 | 0.29549 | 1.558(1.117,2.173) |
| Yes | No | No | Yes | No | 14175 | 30 | 118064.91 | 0.2541 | 1.349(0.931,1.955) |
| Yes | No | No | Yes | Yes | 7112 | 21 | 58700.2 | 0.35775 | 1.947(1.256,3.02) |
| Yes | No | Yes | No | No | 8972 | 22 | 74604.3 | 0.29489 | 1.562(1.017,2.399) |
| Yes | No | Yes | No | Yes | 6274 | 12 | 51818.28 | 0.23158 | 1.242(0.7,2.206) |
| Yes | No | Yes | Yes | No | 16783 | 36 | 139902.72 | 0.25732 | 1.384(0.984,1.947) |
| Yes | No | Yes | Yes | Yes | 13441 | 37 | 111104.07 | 0.33302 | 1.815(1.296,2.542) |
| Yes | Yes | No | No | No | 59302 | 128 | 487801.91 | 0.2624 | 1.5(1.227,1.833) |
| Yes | Yes | No | No | Yes | 51068 | 125 | 415655.65 | 0.30073 | 1.755(1.433,2.15) |
| Yes | Yes | No | Yes | No | 28518 | 47 | 234136.67 | 0.20074 | 1.175(0.867,1.592) |
| Yes | Yes | No | Yes | Yes | 25897 | 64 | 209413.02 | 0.30562 | 1.827(1.4,2.383) |
| Yes | Yes | Yes | No | No | 23042 | 51 | 189508.57 | 0.26912 | 1.567(1.169,2.099) |
| Yes | Yes | Yes | No | Yes | 26817 | 60 | 217483.51 | 0.27588 | 1.624(1.236,2.134) |
| Yes | Yes | Yes | Yes | No | 55339 | 119 | 456082.84 | 0.26092 | 1.532(1.246,1.884) |
| Yes | Yes | Yes | Yes | Yes | 75944 | 168 | 616754.75 | 0.27239 | 1.619(1.348,1.945) |

^a^Endometrial cancer incidence per 1,000 person-years.

^b^Adjusted for age, sex, smoking, alcohol consumption, regular exercise; HR, hazard ratio; CI, 95% confidence interval.

Abbreviations: BP, blood pressure; CI, confidence interval; GLU, fasting blood glucose; HDL-C, high-density lipoprotein cholesterol; HR, hazard ratio; Met, metabolic syndrome; Post-MP, post-menopausal; Pre-MP, pre-menopausal; TG, triglycerides; WC, waist circumference. CI, confidence interval; HR, hazard ratio; Ref., reference.
